# Protocol for Studying Reprogramming of Mouse Pancreatic Acinar Cells to β-like Cells

**DOI:** 10.1016/j.xpro.2020.100096

**Published:** 2020-08-28

**Authors:** Ofer Elhanani, Michael D. Walker

**Affiliations:** 1 Department of Biomolecular Sciences, Weizmann Institute of Science, Rehovot 76100, Israel

## Abstract

The potential of reprogrammed β cells derived from pancreatic exocrine cells to treat diabetes has been demonstrated in animal models. However, the precise mechanisms and regulators involved in this process are not clear. Here, we describe a method that allows mechanistic studies of this process in primary exocrine cultures using adenoviral expression vectors. This rapid 5-day protocol, provides the researcher with a highly controlled experimental system in which the effects of different compounds or genetic manipulations can be studied.

For complete details on the use and execution of this protocol, please refer to Elhanani et al. (2020).

## Highlights

• *In vitro* isolation, infection, and culturing of mouse pancreatic exocrine cells
• PDX1, NGN3, and MafA induce reprogramming of exocrine to β-like cells *in vitro*
• Reprogrammed cells express endocrine genes and produce and secrete insulin
• A rapid highly controlled system for mechanistic studies of β cell reprogramming

## Graphical Abstract

**Figure:**
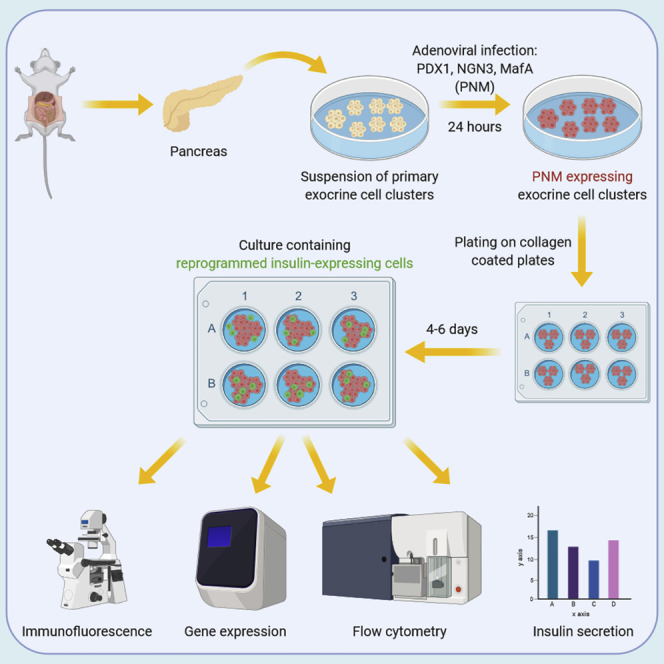

## Before You Begin

Before beginning the protocol, all adenoviral vectors to be used, should be amplified, aliquoted and their titer determined. In addition, collagen-coated plates should be prepared in advance to allow seeding of the cells one day following their isolation. On the day of cell isolation, prior to beginning of the protocol, prepare fresh HBSS wash solution and collagenase P solution and keep them in ice until use.

### Amplification of Adenoviruses

**Timing: 1 week**

To induce reprogramming of exocrine to β cells, adenoviral expression vectors encoding PDX1, NGN3 and MafA (PNM) are used. The combination of the separate vectors, or a single adenoviral vector expressing all three PNM factors (Ad-M3-mCherry, (Li et al., 2014a)) can be used. While the separate vectors generate both β-like (insulin-expressing) and δ-like (somatostatin-expressing) cells, a single vector expressing all PNM factors generates predominantly β-like cells. Hence, for studies aiming to specifically address reprogramming to the β cell lineage, using the single vector is preferable. This step describes the amplification of viruses beginning with the initial primary virus stock that is obtained using a standard protocol for generation of recombinant adenoviral vectors.

1. Plate 2.5 × 10^6^ 293A cells on two 100 mm tissue culture plates and allow to cells to attach for 16–20 h (standard tissue culture incubator: 37°C, 5% CO_2_)^.^
2. On the next day, cells should be 90%–95% confluent. Replace the media and add 100 μL of adenoviral stock to each plate and place in the incubator.
3. Incubate the cells until ∼95% of cells are detached from the plate. This usually occurs after 48 h, but sometimes require longer incubation.
4. Collect the media of both plates into 50 mL polypropylene tube (Falcon).
5. Seal the tube with parafilm (to avoid contaminating the centrifuge with adenoviruses) and centrifuge 600 × *g*, 5 min.
6. Aspirate the supernatant.
7. Resuspend the cell pellet in 2 mL of PBS.
8. Transfer the cell suspension to two Eppendorf tubes and seal with parafilm.
9. Lyse the cells by four cycles of freezing (liquid nitrogen)/thawing (37°C).
10. Centrifuge at max speed for 10 min.
11. Transfer the supernatant to a new tube.
12. Use all volume (2 mL) of obtained virus in step 11, for reinfection in a second round of amplification: follow steps 1–11 again using two 150 mm tissue culture plates, plated with 7.5 × 10^6^ 293A cells each. Infect each plate with 1 mL of virus stock.
13. After the second round of amplification, add to the 2 mL of obtained virus, 400 μL of 50% glycerol (∼10% glycerol final) and vortex.
14. Aliquot in 30–50 μL aliquots. This is the virus stock (ready for use).
15. Store at −80°C.

***Note:*** Follow all safety regulations and guidelines for working with adenoviral vectors. Make sure to wash consumables in bleach before disposal.

***Note:*** To avoid cross-contamination of adenoviral stocks, we strongly recommend not to amplify more than a single adenoviral vector at a time.

### Measurements of Adenoviral Titers

**Timing: 3 days**

In this section, the titers of adenoviral vectors are measured based on staining of the hexon protein, a coat protein found in adenoviruses. Hexon-positive cells mark infected cells and the titer is calculated based on the number of infected cells obtained by using a known dilution factor of the viral stock. Adenoviral vectors containing a reporter such as a fluorescent protein, do not require hexon staining as infected cells can be counted based on reporter expression.

**Figure 1:**
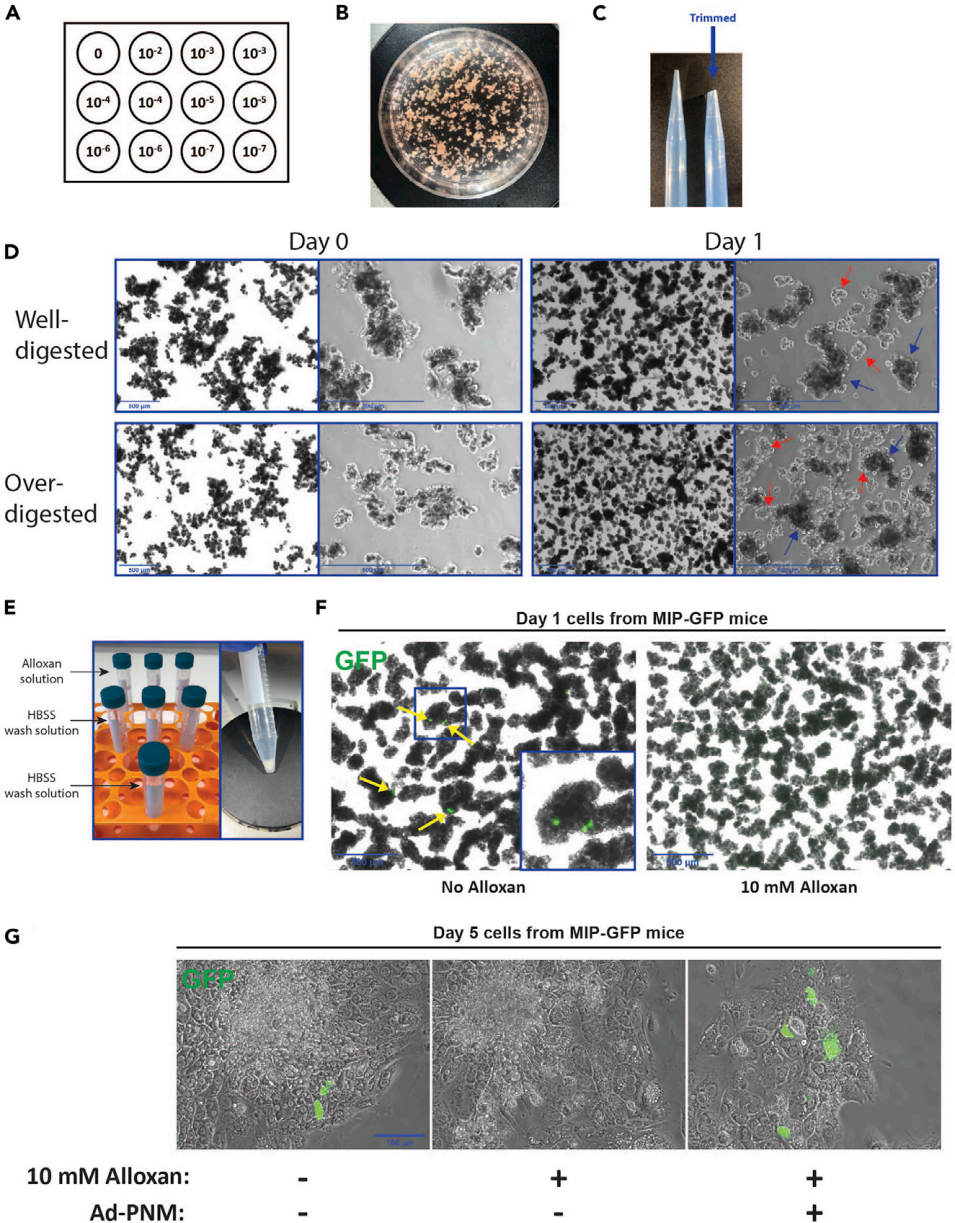
Isolation of Primary Exocrine Cells (A) 12-well plate setup of adenoviral dilutions for titer measurements. (B) Pancreatic tissue minced with scissors. (C) Trimmed 1 mL pipette tip to be used in all stages that require transferring of cells. (D) Exocrine cell-clusters at the end of isolation protocol (day 0) or 24 h following isolation (day 1). During isolation protocol, pancreatic tissue was digested with collagenase P solution for 12 min (well-digested) or for 17 min (over-digested). Blue arrows, exocrine cell clusters. Red arrows, dead cells. Scale bars, 500 μm. (E) 15 mL tubes setup for alloxan treatment followed by washes with HBSS wash solution (left). Exocrine cell clusters pellet following sedimentation by gravity (right). (F) Day 1 exocrine cell clusters isolated from MIP-GFP (mouse insulin promoter GFP) mice treated and untreated with 10 mM alloxan. Yellow arrows, GFP-expressing native β cells appear only in cells untreated with alloxan. Scale bar, 500 μm. (G) Day 5 exocrine cell clusters isolated from MIP-GFP mice treated and untreated with 10 mM alloxan and Ad-PNM to induce reprogramming. Scale bar, 100 μm.
19. Aspirate the virus-containing medium.
20. Fix the cells with 1 mL of cold methanol per well for 10 min.
21. Wash the cells three times using PBS + 1% BSA.
22. Dilute the anti-hexon-HRP antibody 1:500 in PBS+1% BSA
23. Incubate the cells at 37°C for 2 h with diluted antibody.
24. Wash the cells three times using PBS + 1% BSA.
25. Incubate with DAB solution until staining of immunoreactive cells becomes visible (usually after 10–15 min).
26. Count immunoreactive cells under a microscope in at least ten fields of view (use ×10, ×20, or ×40 objective). For counting, we recommend choosing a well with a viral dilution that results in 5–50 stained cells per field of view.
27. Calculate the titer according to the following formula: IFU/mL=(mean cells/field × fields/well)/(0.1 mL × virus dilution)

***Note:*** numbers of fields per well is determined by the magnification of objective used: ×10, 150 fields; ×20, 594 fields; ×40, 2,376 fields.

***Note:*** to measure titers of viruses that express a fluorescent reporter, the staining in stages 5–10 is not required. Fluorescent cells can be counted instead of immunoreactive cells and calculation of viral titer is performed in the same manner.

16. Plate 5 × 10^5^ 293A cells/well on a 12-well tissue culture plate.
17. Prepare serial dilutions of the adenovirus stock to be measured in the range of 10^−2^–10^−7^.
18. Infect each well with 100 μL of diluted virus as described in Figure 1A. Place the plate in a standard tissue culture incubator for 48 h.

### Preparation of Collagen-Coated Plates

**Timing: 1 day**

28. Dilute Type I collagen solution 1:10 in DDW.
29. According to the needs of experiment, choose the desired number and type of tissue culture cell plates/wells. To each well, add the diluted collagen solution. The considerations for choosing the plates needed appear in Step-By-Step Method Details- Plating of cells section, step 6.
30. Seal the plates with parafilm.
31. Incubate for 16–20 h at 4°C.
32. Aspirate collagen solution from all wells (collagen solution can be recycled and used for up to 1 month after dilution).
33. Wash the wells twice with PBS.
34. Allow wells to dry by leaving the plates without the cover in the biological hood for 2–20 h.
35. Use plates immediately, or seal the plates with parafilm, and store at 4°C for up to 2 weeks.

## Key Resources Table

**Table:** 

REAGENT or RESOURCE	SOURCE	IDENTIFIER
**Antibodies**		

guinea pig anti-insulin	DAKO	CAT# A0564; RRID: AB_10013624
goat anti-somatostatin	Santa Cruz biotechnology	CAT# SC-7819; RRID: AB_2302603
rabbit anti-MafA	Bethyl	CAT# IHC-00352; RRID: AB_1279486
goat anti-PDX1	Prof. C. Wright, Vanderbilt University	N/A
rabbit anti-NGN3	Beta Cell Biology Consortium	CAT# AB2011; RRID: AB_10014614
goat anti-Hexon-HRP conjugated	Acris Antibodies	CAT# BP2297HRP; RRID: AB_972522

**Bacterial and Virus Strains**		

Ad-M3-mCherry	(Li et al., 2014a)	Addgene: pAd-M3cherry (plasmid #61041)
Ad-mCherry	Prof. Qiao Zhou, Department of Stem Cell and Regenerative Biology, Harvard University. (Li et al., 2014a)	N/A
Ad-PDX1 (rat)	Prof. Sarah Ferber, Sheba Medical Center, Israel. (Sapir et al., 2005)	N/A
Ad-NGN3 (human)	Prof. Sarah Ferber, Sheba Medical Center, Israel. (Gasa et al., 2004)	N/A
Ad-MafA (mouse)	Prof. Sarah Ferber, Sheba Medical Center, Israel. (Berneman-Zeitouni et al., 2014)	N/A
Ad-LacZ	Prof. Sarah Ferber, Sheba Medical Center, Israel. (Berneman-Zeitouni et al., 2014)	N/A

**Chemicals, Peptides, and Recombinant Proteins**		

collagenase P	Roche	CAT# 11213873001
DAPI	Sigma	CAT# 32670
Alloxan monohydrate	Sigma-Aldrich	CAT# A7413
Trypsin inhibitor from *Glycine max* (soybean)	Sigma-Aldrich	CAT# T9128
murine EGF	PeproTech inc.	CAT# 315-09
Collagen, Type I solution from rat tail	Sigma-Aldrich	CAT# C3867
Paraformaldehyde	Electron Microscopy Sciences	CAT# 15710
Hanks’ balanced salt solution	Sigma-Aldrich	CAT# H6648
FBS	Biological Industries	CAT# 04-007-1A
Waymouth’s MB752/1 culture medium	Biological Industries	CAT# 01-110-1A
L-Glutamine solution (200 mM)	Biological Industries	CAT# 03-020-1B
penicillin-streptomycin solution	Biological Industries	CAT# 03-031-1B
RPMI 1640 medium, no glucose, no glutamine	Biological industries	CAT# 01-101-1A
D-(+)-Glucose solution	Sigma-Aldrich	CAT# G8769-100ML
DMEM, high glucose medium	ThermoFisher scientific	CAT# 41965039
HEPES	Sigma-Aldrich	CAT# H0527
KCl	J.T.Baker	CAT# 3040-19
MgCl2	MERCK	CAT# 105833
EDTA	J.T.Baker	CAT# 8993-01
EGTA	Sigma-Aldrich	CAT# E4378
NP-40	Sigma-Aldrich	CAT# NP40-100ML
PBS ×10	Biological industries	CAT# 02-023-5A
Triton X-100	Sigma-Aldrich	CAT# T8787-100ML
Glycerol	J.T.Baker	CAT# 2136-01
Bio-Rad protein assay	Bio-Rad	CAT# 500-0006
SIGMA*FAST*™ 3,3′-Diaminobenzidine tablets (DAB)	Sigma-Aldrich	CAT# D4293
Bovine serum albumin	Sigma-Aldrich	CAT# A7888

**Critical Commercial Assays**		

Direct-zol RNA miniprep kit	Zymo research	CAT# R2052
RNeasy micro kit	QIAGEN	CAT# 74004
SensiFast cDNA synthesis kit	Bioline	CAT# BIO-65054
Power SYBR green PCR mix	Applied Biosystems	CAT# 4367660
CAS-Block	Life technologies	CAT# 008120
Fixable viability dye eFluor450	Invitrogen	CAT# 65-0863
Insulin ultra-sensitive kit	Cisbio	CAT# 62IN2PEG

**Experimental Models: Cell Lines**		

293A cells	Invitrogen	CAT# R705-07

**Experimental Models: Organisms/Strains**		

Wild-type mouse: C57BL/6JOlaHsd	ENVIGO	C57BL/6JOlaHsd
Transgenic mouse: MIP-GFP	The Jackson Laboratory; (Hara et al., 2003)	JAX: 006864

**Oligonucleotides**		

Primer: ARX forward: CCGCTGGGTCTGAGCACTTT	This paper	N/A
Primer: ARX reverse: AAAGAGCCTGCCAAATGCTGG	This paper	N/A
Primer: Glucagon forward: AGGAACCGGAACAACATTGC	This paper	N/A
Primer: Glucagon reverse: GCCTGGCCCTCCAAGTAAGA	This paper	N/A
Primer: Hhex forward: TCAGAATCGCCGAGCTAAAT	This paper	N/A
Primer: Hhex reverse: GCTCACAGGAAGTGTCCAAA	This paper	N/A
Primer: HPRT forward: ACAAAGCCTAAGATGAGCGC	This paper	N/A
Primer: HPRT reverse: CAACATCAACAGGACTCCTC	This paper	N/A
Primer: INS1 forward: CAGCAAGCAGGTCATTGTTT	This paper	N/A
Primer: INS1 reverse: ACCACAAAGATGCTGTTTGACA	This paper	N/A
Primer: MafA forward: CACATTCTGGAGAGCGAGAA	This paper	N/A
Primer: MafA reverse: CTCGTATTTCTCCTTGTACAGGT	This paper	N/A
Primer: NGN3 (human) forward: GCAATCGAATGCACAACCTC	This paper	N/A
Primer: NGN3 (human) reverse: AGATGTAGTTGTGGGCGAAG	This paper	N/A
Primer: PDX1 (rat) forward: GAAACGTAGTAGCGGGACA	This paper	N/A
Primer: PDX1 (rat) reverse: CTCCTCGCCCGAGGTTA	This paper	N/A
Primer: Somatostatin forward: CCCAGACTCCGTCAGTTTC	This paper	N/A
Primer: Somatostatin reverse: CAGGGCATCATTCTCTGTCT	This paper	N/A

**Other**		

100 μm cell strainer	SPL Life Sciences	CAT# 93100
35 μm cell strainer	Falcon	CAT# 352235
Eppendorf tubes	Eppendorf	CAT# 0030 120.086
24-well plate	Falcon	CAT# 353047
4-well μ slides	ibidi	CAT# 80426

## Materials and Equipment

**Table:** HBSS Wash Solution

Reagent	Final Concentration	Volume for 500 mL (mL)
Hanks’ balanced salt solution		487.5
Fetal bovine serum (FBS)	2.5%	12.5

**CRITICAL:** Prepare fresh on the day of exocrine cell isolation Collagenase P solution

**Table:** 

Reagent	Final Concentration
HBSS wash solution	
Collagenase P	0.7 mg/mL

**CRITICAL:** Prepare fresh on the day of exocrine cell isolation

***Note:*** Prepare 5 mL of Collagenase P solution per dissected pancreas

**Table:** Waymouth’s MB752/1 Exocrine Cell Culture Medium

Reagent	Final Concentration	Amount for 500 mL
Waymouth’s MB752/1 culture medium		477.5 mL
Fetal bovine serum (FBS)	2.5%	12.5 mL
Glutamine	2 mM	5 mL
Penicillin-streptomycin solution	1% (100 units/mL penicillin, 0.1 mg/mL streptomycin)	5 mL
EGF	25 ng/mL	125 μL (from 100 μg/mL stock solution)
Trypsin inhibitor from *Glycine max*	0.3 mg/mL	150 mg

***Note:*** Store for up to one month at 4°C

**Table:** RPMI Medium for Insulin Secretion Assay

Reagent	Final Concentration	Volume for 500 mL (mL)
RPMI medium without glucose		473.4–476.8
Fetal bovine serum (FBS)	2.5%	12.5
Glutamine	2 mM	5
Penicillin-streptomycin solution	1% (100 units/mL penicillin, 0.1 mg/mL streptomycin)	5
EGF	25 ng/mL	0.125 (from 100 μg/mL stock solution)
2.5 M Glucose solution	3–20 mM based on experimental needs	0.6–4

**Table:** DMEM Medium for 293A Cells (for Amplification of Adenoviruses)

Reagent	Final Concentration	Volume for 500 mL (mL)
DMEM high glucose medium		440
Fetal bovine serum (FBS)	10%	50
Glutamine solution	2 mM	5
Penicillin-streptomycin solution	1% (100 units/mL penicillin, 0.1 mg/mL streptomycin)	5

**Table:** Lysis Buffer

Reagent	Final Concentration	Stock concentration	Volume for 50 mL (mL)
HEPES	20 mM	400 mM	2.5
KCl	10 mM	1 M	0.5
MgCl_2_	1 mM	1 M	0.05
EDTA	1 mM	0.5 M	0.1
EGTA	1 mM	0.1 M	0.5
NP-40	0.5%	20%	1.25
DDW			45.1 mL

**Table:** PBST

Reagent	Final Concentration	Volume for 500 mL (mL)
PBS ×10	×1	50
Triton X-100	0.2%	1
DDW		449

**Table:** FACS Buffer

Reagent	Final Concentration	Stock Concentration	Volume for 500 mL (mL)
PBS ×10 (Ca/Mg free)	×1	×10	50
Fetal bovine serum (FBS)	2.5%	100%	12.5
EDTA	2 mM	0.5 M	2
DDW			435.5

## Step-By-Step Method Details

Previous protocols for exocrine cell isolation and culturing (Gout et al., 2013) or isolation and adenoviral infections (Geron et al., 2014) were published. Here, we optimized an *ex vivo* protocol for isolation, adenoviral infection and culturing of primary exocrine cells for up to 8 days. This protocol allows studies of mechanisms underlying acinar to β-cell reprogramming by adenoviral expression of PDX1, NGN3 and MafA (PNM). It can be used to test the effects of various conditions on reprogramming efficiency and cell functionality. Conditions that can be manipulated include chemical/hormonal treatments, adenoviral overexpression or silencing of studied genes, the use of transgenic mouse lines, temporal changes in reprogramming etc. Use of cells isolated from a reporter mouse line for insulin such as MIP- GFP, permits enrichment for reprogrammed cells, as we previously demonstrated (Elhanani et al., 2020). Isolated cells from such a mouse line can also be used to set up a system for high throughput screen of compounds that affect reprogramming efficiency. Furthermore, this protocol can be used for other applications that require pancreatic exocrine cell isolation and their culturing after adenoviral infections.

### Isolation of Primary Pancreatic Exocrine Cells

**Timing: 3 h**

This stage allows the isolation of pancreatic exocrine cell clusters and their infection with adenoviral vectors to induce their reprogramming to insulin- or somatostatin-expressing cells. As we wish to study the formation of newly formed insulin-positive cells, this protocol includes a stage for elimination of native β cells by treatment with the β cell toxin alloxan. This protocol can be used for applications other than studies of β cell reprogramming. In this case, alloxan treatment is not required as native β cells comprise a small fraction of pancreatic cells and will not interfere with most other applications.

1. Sacrifice the desired number of mice (8–12 week old) by cervical dislocation (in accordance with local IACUC guidelines).
2. Place on ice a 6-well plate containing 4 mL of cold HBSS wash solution per well.
3. Dissect the mouse and excise the pancreas into a single well.
4. Wash the tissue in the well and transfer to a new well containing cold HBSS wash solution.
5. Repeat steps 3–4 for the desired number of mice. Do not let tissues stand on ice for longer than 30 min.
6. Transfer one pancreas into 60 mm tissue culture plate containing 5 mL of cold collagenase solution. Mince the tissue with scissors (Figure 1B) and gently transfer to a 50 mL conical tube using 1 mL pipettor trimmed at the end (to reduce shear forces on the cells, Figure 1C). Place the conical tube in ice while processing the remaining pancreata.
7. Incubate for 12 min at 37°C. After the first 4 min of incubation, and every 2 min until the end of the incubation: pipette the tissues gently five times up and down using 1 mL trimmed pipette.

**CRITICAL:** Collagenase digestion is a critical stage as over-digestion of the tissue can impair cell viability (Figure 1D) and reprogramming efficiency. Batch to batch variation in collagenase may affect its activity and the time of digestion may require calibration to optimize viability at the end of the protocol.

8. To each tube (one digested pancreas in each tube), add 45 mL of ice cold HBSS wash solution.
9. Perform two washes by centrifugation of the cells at 200 × *g* for 3 min followed by aspiration of the supernatant and adding an additional 45 mL of HBSS wash solution. After the first wash, the desired number of pancreata can be combined in a single 50 mL conical tube.
10. Gently resuspend the pellet in 5 mL HBSS wash solution using 1 mL trimmed pipette and then add HBSS wash solution to a total volume of 30 mL.
11. To obtain desirable size of cell clusters and to remove clumps of undigested tissue, filter through a standard metal tea strainer followed by filtration through a 100 μm cell strainer.
12. Centrifuge at 200 × *g* for 3 min and aspirate the supernatant leaving ∼3 mL of liquid.
13. Alloxan treatment (Figures 1F and 1G):
a. Prepare 1M stock solution in DDW.

**CRITICAL:** alloxan is unstable in solution, alloxan stock solution must be prepared fresh immediately before use.

b. Dilute alloxan stock solution 1:100 (10 mM) in desired volume of HBSS wash solution.
c. For each pancreas: prepare 3 × 15 mL conical tubes each containing 9 mL of HBSS+alloxan (Figure 1E, left panel).
d. Resuspend the cell pellet in 3 mL HBSS wash solution and transfer gently 1 mL to each of the 15 mL conical tubes containing the alloxan solution.
e. Incubate for 10 min at room temperature (20°–30°C) and allow cell clusters to sediment by gravity (Figure 1E, right panel). This helps to deplete dead cells and enrich for large cell clusters.

***Optional:*** alloxan treatment depletes native β cells from the culture. This step is critical when studying reprogramming of exocrine to endocrine cells. However, as native β cells comprise a small fraction of the cells in the culture, their presence will not interfere with most other application of this protocol. Hence, if this protocol is to be used for other application than reprogramming, use HBSS wash solution without alloxan in this step.

14. Aspirate alloxan solution leaving ∼1 mL at the bottom of the tube. Resuspend the cell pellet with trimmed 1 mL pipette tip and from each tube transfer to a new 15 mL tube containing 9 mL of HBSS wash solution (3 tubes per pancreas, Figure 1E, left panel). Allow cell clusters to sediment by gravity to the bottom of the tube for 7 min.
15. Repeat the last step but this time combine three tubes containing cells from the same pancreas into one 15 mL tube.
16. From this stage, all subsequent steps should be performed in the tissue culture hood. Aspirate the HBSS wash solution from the 15 mL conical tube leaving a minimal volume at the bottom.
17. Resuspend the cells gently using a trimmed 1 mL pipette tip and resuspend in 3 mL of Waymouth’s culture medium in a 50 mL conical tube.
18. Plating of cells: for reproducibility, in every experiment the ratio of virus titer to the number of cells should remain constant. Since cells are clustered, it is impractical to count them. As an alternative protein content is measured and 850 μg protein from cells is plated per well in a 6-well plate:
a. Mix cells gently by swirling the conical tube and immediately transfer 100 μL of cells to an Eppendorf tube.
b. Wash the cells twice with PBS by centrifugation of the cells at 500 × *g* for 4 min.
c. Lyse the cells using 100 μL of lysis buffer.
d. Measure protein concentration using Bio-Rad protein assay.
e. Calculate the protein concentration in the original tube containing cells.
f. Dilute the cells to a final concentration of 1,700 μg protein equivalent /mL with Waymouth’s medium.
g. Add 1.5 mL of Waymouth’s medium to the desired amount of wells in 6-well plates (non-collagen coated at this stage).
h. To each well add 0.5 mL of cell suspension.

**CRITICAL:** exocrine cell clusters sink very fast. While plating, it is important to continually and gently swirl the tube containing the cells in order to uniformly mix them. Otherwise, a non- uniform number of clusters will be plated in each well.

19. Transfer the plates to a standard tissue culture incubator (37°C, 5% CO_2_) while preparing the adenoviruses for infection.

### Adenoviral Infections

**Timing: 20 min**

In this section, exocrine cell clusters isolated in the previous section are infected with adenoviral vectors that induce reprogramming to the endocrine lineages: Ad-PDX1 and Ad-NGN3 induce reprogramming to the somatostatin δ cell lineage. The addition of Ad-MafA to this mix, results in reprogramming to the insulin-expressing β cell lineage. Notice that infection with this virus mix will result in formation of mutually exclusive mono-hormonal cells that express either somatostatin or insulin. Using a single viral vector that expresses all three PNM factors (namely Ad-M3-mCherry) results in formation of predominantly insulin-expressing cells. We provide below the titers we use for infections with each of these viral vectors. However, before initiating use of a new viral vector, it is important to calibrate the desired titer for infection, as different vectors achieve maximal expression at different titers and some vectors compromise cell viability at high titers. See the “Expected Outcomes” section for further details on titer calibration.

20. For each well, prepare a mix that contains the desired volume of virus stocks in 50 μL Waymouth’s medium per well. See Table 1, an example of preparation of virus mix. We use the following final titers (in the 6-well plate containing cells in 2 mL medium):

**Table 1:** An Example for Preparation of Virus Mix of PNM for Infection of 6 Wells

Virus	Virus Stock Titer (IFU/mL)	Final Desired Titer in Each Well Containing 2 mL Medium (IFU/mL)	Volume per Well (μL)	Volume per 6-Well Mix
Ad-rPDX1	1.09 × 10^10^ IFU/mL	10 × 10^6^ IFU/mL	1.84 μL	11.04 μL
Ad-hNGN3	4.58 × 10^10^ IFU/mL	20 × 10^6^ IFU/mL	0.87 μL	5.22 μL
Ad-mMafA	1.58 × 10^10^ IFU/mL	2.5 × 10^6^ IFU/mL	0.32 μL	1.92 μL
Waymouth’s medium	n/a	n/a	46.97 μL	281.82 μL
**Total**	**n/a**	**n/a**	**50 μL**	**300 μL**
a. Ad-rPDX1 10 × 10^6^ IFU/mL (Sapir et al., 2005)
b. Ad-hNGN3 20 × 10^6^ IFU/mL (Gasa et al., 2004)
c. Ad-MafA 2.5 × 10^6^ IFU/mL (Berneman-Zeitouni et al., 2014)
d. Ad-M3-mCherry 20 × 10^6^ IFU/mL (Addgene: pAD-M3cherry, plasmid #61041); (Li et al., 2014a)

***Note:*** To study reprogramming to the δ-cell lineage (somatostatin-expressing cells), we use infection with Ad-hNGN3 alone or together with Ad-rPDX1 (to increase efficiency (Elhanani et al., 2020)). To study reprogramming to the β cell lineage (insulin-expressing cells), we use a mix of Ad-rPDX1, Ad-hNGN3 and Ad-mMafA, or a single adenoviral vector encoding all three PNM factors: Ad-M3-mCherry.

**CRITICAL:** The titer used for each virus should be calibrated separately. See the “Expected Outcomes” section for further details.

21. Vortex the virus mix well.
22. Remove the plate containing the cells from the incubator and add 50 μL of the virus mix to each well. Mix well by swirling the plate gently.
23. Put the plate back into the incubator.

**CRITICAL:** Exocrine cell clusters tend to clump in the middle of the culture plate. To avoid this, open the incubator door, and just before placing the plate inside, swirl it once more in order to evenly distribute the cells in the plate.

24. Incubate the cells for 16–24 h.

### Plating the Cells

**Timing: 1 h**

25. On the next day, collect the cell suspension from each well (using 1 mL trimmed pipette tip) into a 15 mL tube. Cell suspensions from the same experimental group can be collected to a single tube.
26. Allow the cells to sediment by gravity for 8 min to deplete dead cells and aspirate maximum amount of media leaving the cell pellet at the bottom.
27. To wash away the virus, for each experimental group, prepare a new 15 mL tube containing 9 mL of Waymouth’s medium. Collect the cell pellet using 1 mL trimmed pipette tip and gently add the cell suspension to the top layer of media in the new tube.
28. Allow the cells to sediment by gravity for 8 min to deplete dead cells and aspirate maximum amount of media leaving the cell pellet at the bottom.
29. Resuspend the cell pellet in Waymouth’s medium in the desired volume for plating. For volumes larger than 3 mL, we recommend using 50 mL conical tubes to allow proper mixing of the cells by swirling prior to plating.

**CRITICAL:** Do not mix the cells by pipetting them up and down, this can seriously damage the integrity and viability of exocrine cell clusters.

30. Plate the cells on collagen-coated plates according to the desired subsequent application:
a. For RNA extraction: plate the cells on 6-well or 24-well plates. Cells from each infected well (from the original 6-well plate), can be plated on four wells of 24-well plate.
b. For immunofluorescence: plate on 24-well plates, or on 4-well μ slides (ibidi) or on wells containing collagen-coated plastic coverslips. Do not use glass coverslips as cells do not attach well to them.
c. For insulin secretion assay: plate cells on 12-well plates. Cells from each infected well (from the original 6-well plate) should be plated on two wells of 12-well plates
d. For application that require large numbers of cells such as flow cytometry or ChIP, plate cells on 10 cm culture dishes. All cells from a 6-well plate (from the original 6-well plate) should be plated on one 10 cm culture dish.

**CRITICAL:** exocrine cell clusters sink very quickly and this may result in an uneven distribution of the number of cells between wells. To avoid this, during plating, the tube containing the cells should be continuously mixed by gentle swirling.

***Optional:*** exocrine cell clusters can also be grown in suspension. If cells are plated on non-collagen-coated plates most will remain in suspension. In our hands, this did not affect reprogramming efficiency.

31. Transfer the plates to a standard tissue culture incubator (37°C, 5% CO_2_).
32. After 48 h change the medium: most cell clusters will attach to the plate. Aspirate the old medium containing dead cells and unattached cell clusters and add fresh medium.
33. Medium should be changed every other day.
34. Endocrine gene expression is already detected 3 days post cell isolation. Insulin-positive cells will begin to appear 5 days after cell isolation. Cells can be cultured for up to 8 days, after which they lose viability.

### Assessing Reprogramming: Endocrine Gene Expression Analysis

**Timing: 4 h**

To analyze efficiency of the reprogramming protocol, endocrine gene expression can be measured by qPCR. Endocrine gene activation is detected 3 days following isolation of the cells. For control, wells infected with a mock adenovirus such Ad-mCherry (Li et al., 2014a) or Ad-LacZ (Berneman-Zeitouni et al., 2014) are used.

35. To analyze gene expression, cells are harvested with Tri-reagent at the desired time point and RNA isolation and cDNA synthesis is performed using standard protocols.

**CRITICAL:** isolation of intact RNA from exocrine cells is often challenging due to high expression of digestive enzymes. Cultured exocrine cells lose enzyme gene expression with time in culture (Rooman et al., 2000). Therefore, RNA extracted after 3 days in culture will typically be intact. If analysis of RNA at earlier time points is required, RNA integrity should be thoroughly assessed by gel or tape-station.

36. Perform qRT-PCR with desired primers for endocrine genes.

### Assessing Reprogramming: Hormone Production

**Timing: 4 h**

Hormone production is analyzed by immunofluorescent staining of insulin and somatostatin. Hormones will be detected 5 days following exocrine cell isolation.

37. Aspirate the medium and fix cells for 12 min in 4% PFA
38. Perform three washes of the cells with PBST (PBS supplemented with 0.2% Triton X-100). In the third wash, leave the cells in PBST for 15 min before continuing with the protocol. All subsequent washes are performed in PBST.
39. For blocking, incubate the cells for 1 h with CAS-Block.

***Optional:*** supplement CAS-Block with 5% horse serum during blocking.

40. Dilute primary antibodies in CAS-Block: guinea pig anti-insulin (DAKO, A0564, 1:300) and goat anti-somatostatin (Santa Cruz Biotechnology, SC-7819, 1:200).
41. Incubate with primary antibodies for 1–3 h at room temperature (20–30°C) or overnight (16–20 h) at 4°C.
42. Wash the cells three times with PBST
43. Incubate with secondary antibodies (Jackson ImmunoResearch) diluted 1:200 in CAS-Block for 1 h at room temperature (20–30°C).
44. Wash the cells three times with PBST
45. Stain nuclei for 10 min using 1 μg/mL DAPI in DDW.
46. Wash DAPI with PBS.
47. If using 24-well plates or ibidi 4-well μ slides, cells can be directly imaged. Alternatively, if using plastic coverslips, mount on slides before imaging.

### Flow Cytometry

**Timing: 1 day**

**Figure 2:**
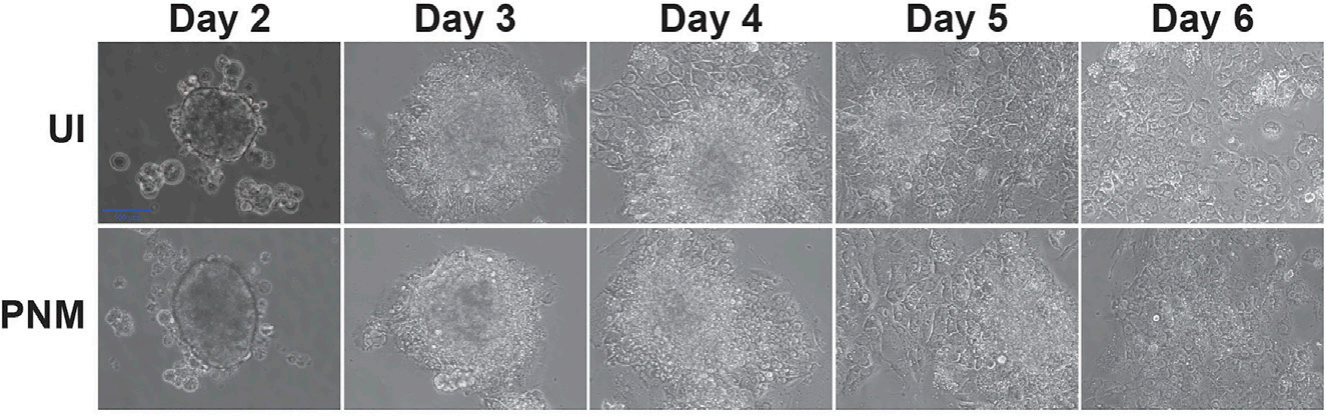
Daily Morphology of Exocrine Cell Clusters Uninfected (UI) or PNM Infected Exocrine cell clusters attached to collagen-coated become flatter with time of culturing and reach monolayer at day 6. No significant morphological differences are observed between uninfected and PNM-infected cells. Scale bar, 100 μm.

Analysis of gene expression and hormone production as described in the previous sections can also be performed by Fluorescence-activated cell sorter (FACS) or analyzer. To analyze gene expression of PNM- expressing cells, Ad-M3-mCherry-infected cells, are sorted based on mCherry signal. By using exocrine cell clusters isolated from MIP-GFP mice (mouse insulin promoter-GFP, (Hara et al., 2003)), a population enriched for reprogrammed cells can be obtained based on GFP expression, as we previously described (Figures 2A, S2B, and S2E in Elhanani et al., 2020). To analyze hormone production by flow cytometry, cells are dissociated, fixed and internally stained for hormone expression.

48. Aspirate culture medium following desired time of culture.
49. Wash the cells once with PBS.
50. Treat cells with 0.025% trypsin (Biological Industries, 03-054-1B, diluted 1:2 in PBS) for 12 min in 37°C.
51. Following incubation, dissociate the cells by pipetting up and down 10–15 times using 1 mL pipette tip.
52. Transfer the cell suspension to Eppendorf tubes containing 100 μL FBS (for quenching of trypsin).
53. Centrifuge the cells for 5 min at 500 × *g* and wash twice with FACS buffer (PBS, 2 mM EDTA, 2.5% FBS).
54. Centrifuge again for 5 min at 500 × *g* and aspirate FACS buffer.
55. Resuspend the cell pellet in fixable viability dye eFluor450 diluted 1:1,000 in FACS buffer to label dead cells.

**CRITICAL:** The expected cell viability is 50%–75%. As a result, staining for dead cells is necessary to obtain reliable flow cytometry results. This step cannot be omitted from the protocol.

56. Incubate for 30 min on ice.
57. Wash the cells once by centrifugation at 500 × *g* for 5 min and resuspend in FACS buffer.
58. If cell-sorting is required for gene expression analysis:
a. Pass the cell suspension through a 35 μm cell strainer to deplete cell clumps.
b. At this stage cells are taken to the FACS instrument. Gate the living cells based on fixable viability dye eFluor450 stain (Figure 4D).
c. Sort desired populations of cells directly into lysis buffer supplemented with RNA- extraction kit. We recommend the RNeasy micro kit.
d. Proceed with RNA extraction and follow standard protocols for qPCR or library preparation for RNA-sequencing.
59. If analysis of hormone production with intracellular stain is required:
a. Fix the cells in 4% PFA in PBS for 10 min.
b. Centrifuge at 600 × *g* for 5 min and resuspend cell pellet in PBST.
c. Follow the protocol for immune-staining in the previous section with 600 × *g*, 5 min centrifugations between stages.
d. Pass the cell suspension through a 35 μm cell strainer to deplete cell clumps.
e. Take the cells to the flow cytometer, gate the living cells based on the fixable viability dye eFluor450 stain (Figure 4D) and analyze the data.

### Assessing Reprogramming: Insulin Secretion

**Timing: 4 days**

To measure insulin secreted by reprogrammed cells (typically ∼3% of the cells in the culture), the cumulative secretion between days 5 to 8 is measured. Since cell culturing for this time frame is required to obtain detectable amounts of insulin, this assay is performed in complete RPMI culture media rather than the standard Krebs Ringer buffer usually used in insulin secretion assays. Cell lysates are also collected to allow measurement of insulin content. This assay is performed on cells plated on 12-well plates.

60. Aspirate the medium (Waymouth’s) of day 5 cells.
61. Wash the cells once with RPMI medium (supplemented with 2.5% FBS, 2 mM glutamine, 1% penicillin-streptomycin solution and 25 ng/mL murine EGF, and the desired glucose concentrations) and aspirate.
62. To each well, add 300 μL of RPMI medium. At this stage, cells in different wells can be exposed to different treatments (i.e., different glucose concentrations, hormone/compound treatments etc.)
63. Place the plates in the incubator for 3 days.
64. On day 8, collect all medium from each well into Eppendorf tubes.
65. Centrifuge at maximum speed for 10 min and transfer the supernatant to new Eppendorf tubes or to 96-well plates. Discard the pellets. These samples contain the secreted insulin. Insulin can be measured on the same day, or samples can be kept at −20°C.
66. Add 1 mL of PBS supplemented with 2.5% FBS to each well, harvest the cells using a cell scraper and transfer the cell suspensions into Eppendorf tubes.

***Note:*** following harvest, exocrine cells do not pellet well after centrifugation. To improve this, we recommend adding 2.5% FBS to the PBS and to specifically use Eppendorf tubes rather than alternate brands (Eppendorf, #0030 120.086).

67. Centrifuge cells at 600 × *g* for 5 min.
68. Aspirate all supernatant and discard it.
69. Resuspend the cell pellet using 30 μL lysis buffer.
70. Measure insulin both in the supernatant and cell lysates using Insulin ultra-sensitive kit according to manufacturer’s instructions.
71. For normalization, measure the protein content in the cell lysates using Bio-Rad protein assay.
72. Calculate the total amount of secreted insulin, insulin content in cell lysates and protein content in lysates. Normalize secreted and content of insulin to total protein.

## Expected Outcomes

### Cell Isolation and Viral Infections

This protocol typically yields a sufficient number of cells to plate 1–2 6-well plates, from one mouse (5–10 mg protein of cells). If yields are lower, collagenase digestion was probably insufficient and incubation time of the tissue with collagenase can be extended. If larger amounts of cells are obtained at the end of the protocol, this is usually an indication that the tissue was over-digested with collagenase. This will also result in smaller exocrine cell clusters (Figure 1D). In our experience, over-digestion of the tissue with collagenase and plating of small exocrine clusters seriously compromises exocrine cell viability. Therefore, it is important to calibrate the conditions of collagenase digestion to the yields reported here.

To specifically study newly formed, reprogrammed insulin-positive cells, native pancreatic β cells are depleted by alloxan treatment (Figure 1F). In the absence of alloxan treatment, native β cells will survive culturing and will be indistinguishable from newly formed reprogrammed β cells (Figure 1G), hence alloxan treatment is necessary in this protocol.

Attachment of the cells to collagen-coated plates will occur 48 h after plating. At that point, cells will still appear as clusters under the microscope, but cell clusters will become flatter with the time of culture and will appear as a monolayer at around day 6 with no significant morphological differences between uninfected and PNM-infected cells (Figure 2). Primary exocrine cells are hard to culture while preserving viability. In addition to flattening of the cells, cell death will also be observed daily, therefore it is important to replace the medium every two days.

**Figure 3:**
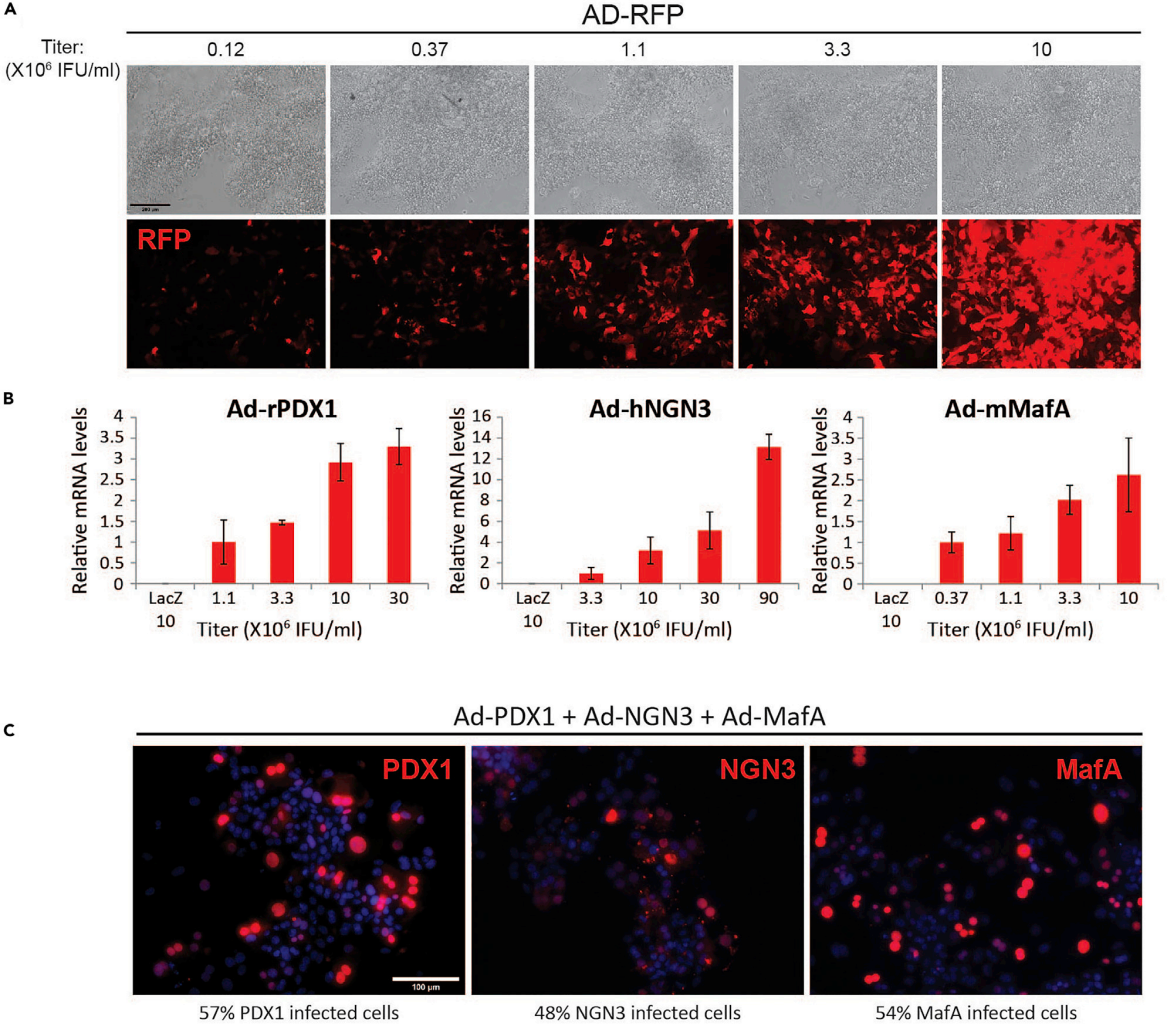
Dependency of Adenoviral Infection Titer and Expression Levels and Efficiency (A) Day 4 exocrine cells infected with Ad-RFP (red fluorescent protein) at indicated titers. Scale bar, 200 μm. (B) Day 3 exocrine cell clusters were infected with either Ad-PDX1 (rat, rPDX1), Ad-NGN3 (human, hNGN3) or Ad-MafA (mouse, mMafA) at indicated viral titers. For negative control, cells were infected with Ad- LacZ at 10 × 10^6^ IFU/mL. The expression levels of PDX1, NGN3 and MafA were analyzed by qRT-PCR. HPRT was used as a reference house-keeping gene. Expression was normalized relative to values obtained with infection at the lowest titer used, which was defined as 1. Data are presented as mean ± SD; n = 3. (C) Day 4 exocrine cells were infected with Ad-PDX1 (10 × 10^6^ IFU/mL) Ad-NGN3 (20 × 10^6^ IFU/mL) and Ad-MafA (2.5 × 10^6^ IFU/mL) and were stained for these factors (antibody dilution factors: goat anti-PDX1, 1:800; rabbit anti-NGN3, 1:50; rabbit anti-MafA, 1:100). The percentage of cells expressing each, was calculated. n: PDX1, 2333 cells from 7 mice; NGN3, 584 cells from 2 mice; MafA, 2,009 cells from 6 mice. Scale bar, 100 μm.

The efficiency as well as extent of transgene expression is heavily dependent on the viral titer used for infection (Figure 3A). We note that different adenoviral vectors produce the highest efficiency and expression levels with different titers, hence calibration by testing expression levels of each virus at different titers is necessary. We strongly recommend choosing the minimal titer that gives maximal expression without affecting cell viability (evident by significant reduction in attachment of cell clusters to collagen-coated plates: <50% of the number of clusters attached in uninfected cells). We tested the expression of PNM in exocrine cells infected with Ad-rPDX1, Ad-hNGN3 or Ad-mMafA at different titers. Notice that pattern of expression is very different between different vectors (Figure 3B). PDX1 expression reached maximal levels at 10 × 10^6^ IFU/mL, and we chose this titer for our protocol. MafA reached its maximal levels at 3.3 × 10^6^ IFU/mL, but at these levels we also observed compromised viability of the cells. Therefore, we used 2.5 × 10^6^ IFU/mL of Ad-mMafA for our reprogramming protocol. Finally, NGN3 levels continued to rise even when titer was increased to 90 × 10^6^ IFU/mL. Since we did not wish to have a large excess of the NGN3 virus over PDX1 and MafA, we used 20 × 10^6^ IFU/mL for reprogramming. Well calibrated titers normally yield infection efficiency of 45%–60% in primary exocrine cells. Notice that despite the different titers used for these viruses, similar infection efficiencies were obtained (Figure 3C).

### Endocrine Gene Expression Analysis

**Figure 4:**
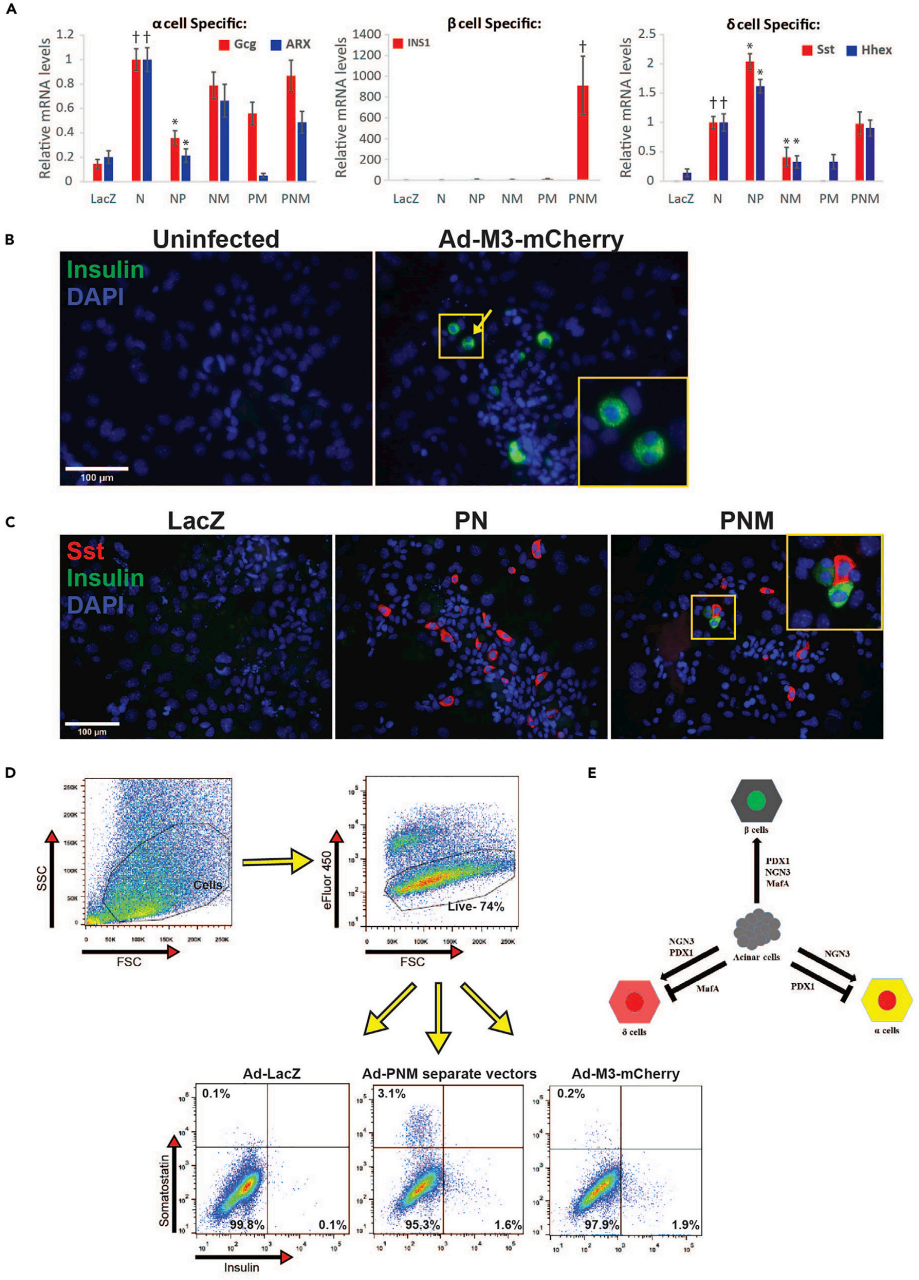
Analyzing Reprogramming (A) Pancreatic exocrine cells were infected with Ad-LacZ control or indicated combinations of Ad-PDX1 (P), Ad-NGN3 (N), Ad-MafA (M). Day 5 cells were analyzed by qRT- PCR for indicated α, β and δ cell-specific genes. HPRT was used as a reference house-keeping gene. Relative expression was calculated relative to the expression level obtained from the Ad-NGN3 infected group = 1. Data are presented as mean ± SEM (n = 5–7; ∗p < 0.05 compared with Ad-NGN3 infected group. †p < 0.05 compared with Ad-LacZ infected group). (B) Exocrine cells were either uninfected or infected with Ad-M3-mCherry and were fixed and stained for insulin 8 days following isolation. Yellow arrow, insulin-expressing binucleated cell. Scale bar, 100 μm. (C) Exocrine cells were infected with LacZ control, PDX1 and NGN3 (PN) or with PDX1, NGN3 and MafA (PNM). Cells were fixed stained for insulin and somatostatin 8 days following isolation. Scale bar, 100 μm. (D) Flow cytometry analysis. Exocrine cells infected with LacZ control, PNM separate vectors or the Ad-M3-mCherry single vector. Day 6 cells were dissociated, stained with fixable viability dye eFluor450, fixed and stained for insulin and somatostatin. Gating of cells on side scatter (SSC) versus forward scatter (FSC) plot, was performed to eliminate debris and large cell clumps (top left). Subsequently, living cells negative for Efluor450 were gated (top right). Population frequencies as defined by somatostatin and insulin expression (bottom). (E) A scheme for the relative contribution of each of the PNM factors to reprogramming to each of the endocrine cell lineages.

Following infection with the PNM vectors, endocrine gene activation is detected as early as 3 days following cell isolation. As an example, we infected exocrine cells with different combinations of the PNM factors and assessed lineage-specific endocrine gene activation in day 5 cells (Figure 4A). Consistent with our previously published results (Elhanani et al., 2020), NGN3 was sufficient to induce the δ cell genes Sst and Hhex. This induction was inhibited by coexpression with MafA and potentiated by PDX1. In contrast, α cell genes ARX and Gcg were induced by NGN3, but inhibited by PDX1 expression (glucagon protein was never detected) and β cell genes such as INS1 were only induced upon coinfection with PDX1, NGN3 and MafA. The relative contributions of each of the PNM factors to different endocrine lineages, is illustrated (Figure 4E). Different combinations of viral vectors can be used depending on the endocrine lineage studied and the biological question.

### Hormone Production

Insulin or somatostatin protein will be detected 5 days following cell isolation. Typically, day 6 Ad-M3- mCherry-infected cells are expected to contain ∼3% newly formed insulin-positive cells (∼6% of infected cells). Infection with the separate adenoviral vectors results in similar reprogramming efficiencies. Ad-PDX1+Ad-NGN3 infected cells are expected to result in ∼8% of somatostatin-positive cells. These numbers increase slightly with time of culture.

As an example, we stained for insulin day 8 cells infected with Ad-M3-mCherry (Figure 4B). Insulin- expressing cells were induced in PNM-expressing (Ad-M3-mCherry infected) exocrine cells. Notice that some of the induced insulin-expressing cells are binucleated, a hallmark of some acinar cells, indicative of the acinar origin of these cells. We also stained control, PDX1 +NGN3 and PDX1 + NGN3 + MafA- expressing day 8 cells for insulin and somatostatin. Whereas PDX1 + NGN3 resulted in appearance of only somatostatin-positive cells, infection with all three PNM factors resulted in expression of somatostatin and insulin in mutually exclusive cells (Figure 4C).

We have previously shown that MafA-positive cells do not induce somatostatin expression (Figure S1E in Elhanani et al., 2020). Here, we used flow cytometry and intracellular staining for somatostatin and insulin to stain PNM-expressing cells infected with the separate viral vectors for PDX1, NGN3 and MafA, or a single viral vector expressing all PNM factors (Ad-M3-mCherry). While infection with the separate vectors induced both somatostatin- and insulin-expressing cells, infection with Ad-M3-mCherry resulted in the induction of predominantly insulin-expressing cells (Figure 4D). This is because unlike with separate PNM vectors, in Ad-M3-mCherry-infected cells, all NGN3-expressing cells also express MafA which blocks the δ cell lineage (Figures 4A and 4E), (Elhanani et al., 2020; Li et al., 2014b).

### Insulin Secretion

Since only 3% of cells in the culture express insulin protein, accumulated insulin secreted over 3 days is measured in the medium to allow detection. In addition, it is important to measure insulin using a sensitive method, hence we chose HTRF (Cisbio, #62IN2PEG). A significant induction of insulin secretion and content is observed in cells infected with Ad-M3-mCherry (Figures 1D and S1A in Elhanani et al., 2020). As we previously demonstrated, these values can be potentiated with GLP-1 receptor agonist (liraglutide) treatment from the 1^st^ day after cell isolation. Although reprogrammed cells exhibit induction of many endocrine and β cell genes, they are immature as indicated by lack of glucose responsiveness. Hence, this protocol can be also be used to study compounds that improve maturity of the cells and would promote glucose-stimulated insulin secretion.

## Limitations

• Cell viability and prolonged culturing: viability of primary exocrine cell cultures is limited. During the isolation protocol and also during culturing, cell death will be observed. To minimize this, it is important to handle the cells gently. Do not over-digest the tissue with collagenase, always use a trimmed pipette when transferring cells, minimize using centrifugations and mix the cells only by gentle swirling. Never vortex or pipette up and down to mix. These will seriously impair cell viability and also reprogramming efficiency. Extended culturing of exocrine cells for 10 days was reported by plating on scaffold matrix such as matrigel (Gout et al., 2013). However, we did not test this reprogramming protocol under these conditions. Viability considerations limit studies on kinetics and maturation of reprogrammed cells to the time frame of up to 8 days.
• Efficiency of reprogramming: as with other reprogramming protocols, this protocol results in relatively low conversion rates. This protocol results in ∼3% of cells in the culture that are positive for insulin protein. This limits the studies to assays that can detect changes in small populations. To overcome this, it is possible to enrich samples for reprogrammed cells using flow cytometry of cells isolated from reporter mouse lines as we have shown using the MIP- GFP mouse (mouse insulin promoter-GFP, (Hara et al., 2003)) (Elhanani et al., 2020).
• Maturity of reprogrammed cells: the cells generated in this protocol are functionally immature as indicated by lack of glucose responsiveness (Elhanani et al., 2020). A possible explanation for this is the limited culturing time mentioned above. Hence, this protocol is suitable to study mechanisms in early stages of reprogramming or treatments that can induce maturity of the cells. Furthermore, as was shown in another system for reprogramming in vitro (Baeyens et al., 2009), maturation of these cells can be studied following their transplantation to mice.

## Troubleshooting

### Problem 1

Cells do not attach well to collagen-coated plates

### Potential Solution

This is usually a problem of viability of the cells. Make sure handling of cells is done gently and centrifugation of exocrine clusters is <200 × *g*. We also observed that over-digestion with collagenase will affect cell viability and attachment to the plates (Figure 1D). Try to reduce the time of collagenase digestion. In addition, in some other protocols, BSA instead of FBS is added to HBSS used in the isolation protocol. In our hands, replacing BSA with 2.5% FBS improved cell viability and their attachment to collagen-coated plates. Furthermore, acinar cells produce large amounts of digestive enzymes including proteases which can potentially affect cell attachment. Make sure that trypsin inhibitor is added to the culture media, as indicated in the protocol.

### Problem 2

Lack of appearance of hormone-positive cells

### Potential Solution

Cell viability is extremely important for this issue as well. All suggestions indicated in problem 1 should be followed to solve this issue as well. Most important is the issue of collagenase digestion. Although over-digestion results in improved yields, it produces small exocrine cell clusters that display impaired reprogramming efficiency. In addition, it is important to optimize the virus titers used for infections. Try to use virus titers that result in high expression without affecting cell viability. Finally, when analyzing insulin-positive cells using immunofluorescence, we noticed batch to batch variation in the background staining of the antibody in acinar cells. As reprogrammed cells produce lower amounts of insulin as compared to native beta cells, not every antibody that works well for staining of islets, will work well for staining reprogrammed cells. Make sure the batch of antibody used produces low background in exocrine cells.

### Problem 3

Low titer of virus stocks after amplification

### Potential Solution

There is variation among different adenoviral expression vectors in yields obtained after amplification. We correct for this by adjusting the volume of virus used for infection of exocrine cells.

### Problem 4

Cells do not pellet well after centrifugation in microcentrifuge tubes.

### Potential Solution

Specifically use Eppendorf tubes (Eppendorf, #0030 120.086).

## Resource Availability

### Lead Contact

Further information and requests for resources and reagents should be directed to and will be fulfilled by the Lead Contact, Michael Walker (m.walker@weizmann.ac.il).

### Materials Availability

Reagents generated in this study are available from the Lead Contact with a completed Materials Transfer Agreement.

### Data and Code Availability

This protocol does not include newly generated datasets or codes.
